# Spatiotemporal variability of soil nutrients and the responses of growth during growth stages of winter wheat in northern China

**DOI:** 10.1371/journal.pone.0203509

**Published:** 2018-12-04

**Authors:** Baowei Su, Gengxing Zhao, Chao Dong

**Affiliations:** 1 College of Resources and Environment, Shandong Agricultural University, Tai’an, China; 2 College of Information Science and Engineering, Shandong Agricultural University, Tai’an, China; Huazhong Agriculture University, CHINA

## Abstract

Studying soil nutrient variability and its effect on the growth and development of crops under a traditional tillage mode is the foundation for comprehensively implementing precision agriculture policies at the field scale and ensuring excellent crop management. In this paper, a 28.5 hm^2^ winter wheat field under the traditional cultivation model in Tianzhuang town of Huantai County was selected as the research area. The mesh point method was utilized for sampling (60×60 m), and the characteristics of soil available nitrogen (AN), available phosphorus (AP) and available potassium (AK) variations in the before sowing, reviving, jointing, and filling stages of winter wheat were analyzed using geostatistical and GIS methods. Moreover, Pearson correlation analysis was used to study the response of wheat growth and development to soil nutrient variations. As the growth stages progressed, 1) each nutrient showed the characteristics of low-high-low and moderate variability. The highest AN and AK contents were found at the reviving stage, while AP reached a turning point at the jointing stage. The order of variability of each nutrient was AN>AP>AK. 2) The nutrient variations first increased and then decreased and showed medium to strong spatial correlation. The three nutrients were strongly spatially correlated in the before sowing stage and moderately spatially correlated during the reviving stage. During the jointing and filling stages, AN had moderate spatial correlation, and AP and AK had strong spatial correlation. The spatial correlation of each nutrient was the weakest in the reviving stage, and the spatial correlation of AN was strongest in the before sowing stage, while the spatial correlations of AP and AK were strongest in the jointing stage. The spatial correlation of each soil nutrient decreased from the before sowing stage to the reviving stage and from the jointing stage to the filling stage, and the spatial correlation increased from the reviving stage to the jointing stage. 3) The soil nutrient content first increased and then decreased, and the grades of the nutrients gradually decreased. 4) The correlation between soil nutrients and wheat growth gradually increased. AN had the highest correlation with wheat growth, followed by AK and AP. The effect of soil nutrients on the growth of wheat at the reviving stage was greater than the effect of nutrients in the current stage. The growth of wheat at the jointing stage was mainly influenced by nutrients in the current stage, while the growth of wheat at the filling stage was influenced by the nutrient contents of both the previous and current stages. Thus, the date of fertilizer supplementation should be postponed properly. In this study, the soil nutrient dynamics and their influence on the growth of wheat during the winter wheat growth period under the traditional field model were well described, and these results could provide a theoretical basis for the precision management of soil nutrients in the northern winter wheat area where the planting environment and cultivation management are relatively uniform.

## Introduction

Research on soil and crops under field conditions is a key step in advancing precision agriculture from theory to practice. Soil continuously varies in time and space and is the carrier and nutrient source for plant growth [1–3]. The basis for precision management and fertilization of farmland is the understanding of the temporal and spatial variability in soil nutrients and the crop responses in different growth stages in time [4–7].

Soil nutrient variability has long been a research hotspot for scholars throughout the world [8–10], and many experiments have been carried out on various nutrients and at different scales. For example, Duraisamy et al. [11] studied the spatial heterogeneity of soil pH, organic carbon and available nutrients in the typical dry areas of India. Rosemary et al. [12] studied the spatial structure of topsoil at multiple scales and land use intensities. Paz et al. [13] explained that soil nutrients and trace elements exhibited spatial dependence even in very small areas (approximately 1.8 hectares). Yang et al. [14] compared the scale effects of soil available phosphorus (AP) and available potassium (AK) on spatial variability under different sampling scales. Blanchet et al. [15] analyzed the effects of different land use patterns, soil types, topographies and other factors on the spatial variability of soil K. However, in general, most studies have focused on the spatial variability of soil nutrients, with less consideration of the temporal variability and their effects on crop growth. Thus, the spatiotemporal variability of soil nutrients in different crop growth stages remains unclear [16–18].

Santillano-Cázares et al. [19] showed that the temporal variability of soil nutrients was much greater than its spatial variability. Qiao et al. [20] studied the characteristics of the temporal and spatial variations in soil in wheat fields that used drip irrigation for different numbers of years. However, these studies are still limited to the analysis of soil nutrients themselves, and the research on soil nutrient variability combined with crop development is still very scarce. Plant growth depends on the absorption of soil nutrients, so soil nutrient variability should also cause spatiotemporal variability in plant growth status [21–24]. An analysis of the variability in soil nutrients and its correlation with crop growth will help to clarify the spatiotemporal variability of the variables in a farmland system and have positive impacts on production [25–27]. Meanwhile, we found that previous studies in this area mostly used field experiment methods. Although these methods can obtain better test results by controlling the test conditions, the conclusions have great limitations in the practical application of agricultural extension because the conditions are different from the real field conditions.

Based on this, the characteristics of the variations in soil nutrients and their effects on wheat growth were analyzed by investigating the traditional cultivation management model for the main winter wheat production areas in China to determine the drawbacks of this model and provide a scientific basis for fertilization in different growth stages of wheat.

## Materials and methods

### Ethics statement

The research area of this study is located in Tianzhuang town, Huantai County. All necessary permits for the described field study have been obtained from Huantai agricultural bureau. The land accessed is not privately owned or protected. The field study did not involve endangered or protected species. The study did not involve animal husbandry or experimentation.

### Study area

The study was conducted in Huantai County, which has an area of 28.5 hm^2^ and is located in north-central Shandong Province, China (37°1.98′~37°2.28′N, 117°59.7′~118°0.12′E). The study area is located in the Huanghuai winter wheat region, which is the most important wheat-producing area in China. The region was classified as having a continental monsoon climate in a warm temperate zone. The climate is mild, and the four seasons are distinct. The average annual air temperature is 12.5°C, and the frost-free period lasts 197 days. The annual average precipitation is 587 mm, which mainly falls in 6~9 months. The terrain is flat with an elevation of 5.7 to 6.8 m a.s.l. The main soil types are cinnamon soil (typic udivitrands), and the winter wheat variety is Jimai 17. The entire process followed the traditional cultivation management mode of the farmers. The main points of the process are as follows: traditional plowing mode was adopted, and the plow depth was 20 cm; basal compound fertilizer [N + P_2_O_5_ + K_2_O < 45% (15-15-15)] was applied before sowing on October 12, 2016, at a dosage of 360 kg/hm^2^, sowing was planted by equal row spacing (15 cm), and the sowing depth was approximately 5 cm; urea [CO (NH_2_)_2_] was applied in the reviving stage on March 5, 2017, at a dosage of 300 kg/hm^2^. The winter wheat was sowed on October 13, 2016, and harvested on June 10, 2017, for a total growth period of 260 days.

### Soil sampling and field survey

**Fig 1:**
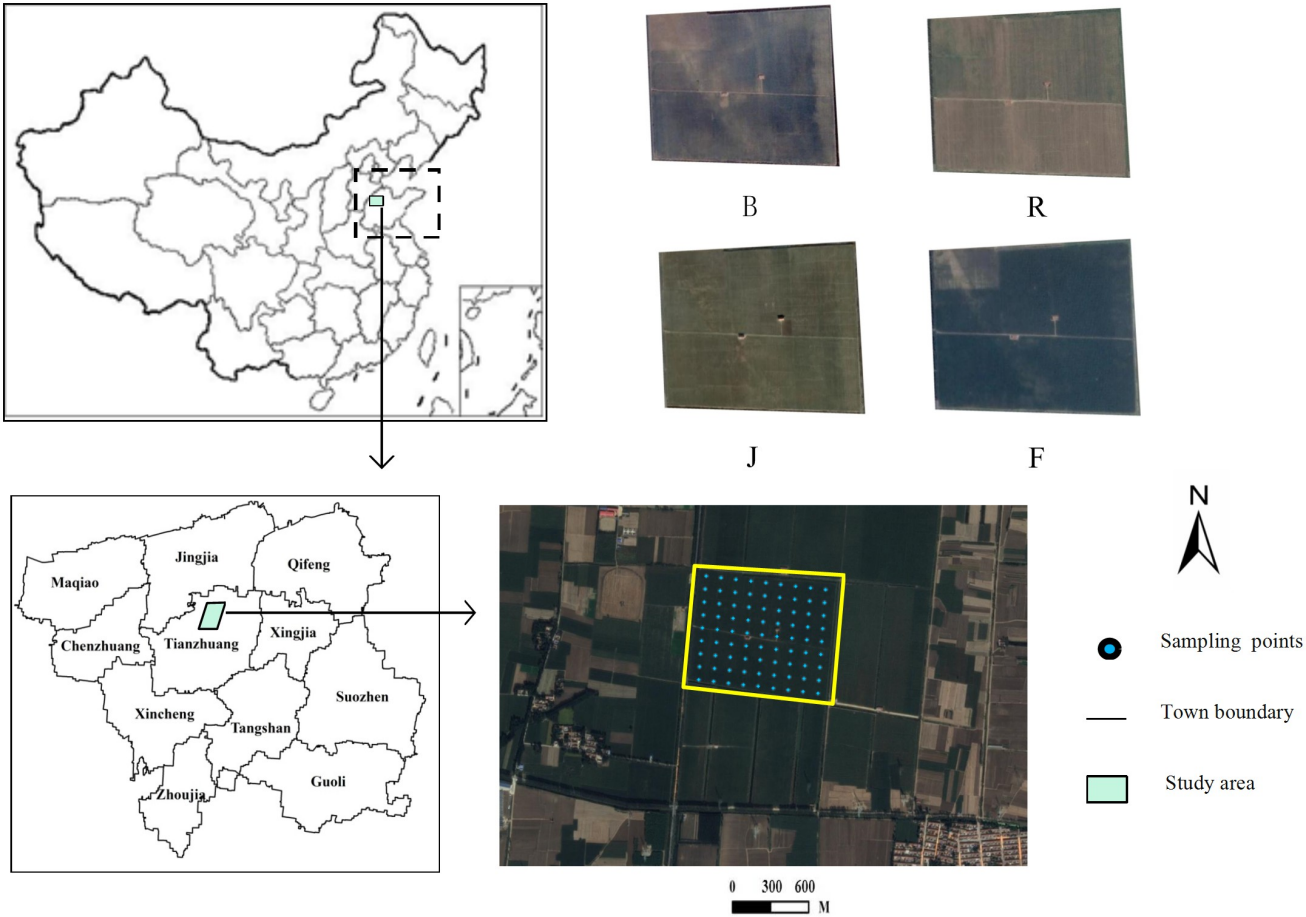
Location of the study area and distribution of soil sampling sites. Note: B: before sowing; R: reviving stage; J: jointing stage; F: filling stage.

First, we collected the basic land use status, soil and topographic maps for Huantai County and obtained the boundary of the study area. Second, a total of 81 sampling points were established according to the standard of uniform grid layout of 60 m×60 m (Fig 1). The coordinates of the sampling points were transformed and input into a Trimble Geo 7x series Global Position System (GPS), which has a positioning accuracy that can reach the centimeter level. Field surveys were conducted on October 8, 2016 (before sowing), March 10, 2017 (reviving stage), April 10, 2017 (jointing stage), and May 15, 2017 (filling stage).

Soil samples were collected by a five-point cross-sampling method with a sampling depth of 0–20 cm. Mixed soil samples from multiple points were brought back to the laboratory, dried, ground and sieved at 2 mm. Soil available nitrogen (AN) was determined by the method of Cornfield [28]; available phosphorous was estimated by the method of Olsen, Cole [29]; available potassium was extracted by 1 mol/L ammonium acetate (pH = 7.0) and determined by the method of Schollenberger and Simon [30]. The chlorophyll contents (SPAD) of the wheat leaves were measured by a Minolta SPAD-502 chlorophyll content analyzer (Spectrum Technologies, Inc.). Five wheat plants were randomly selected at each observation point, and three measurements per plant were collected. The average value of the five wheat plants was calculated as the SPAD value for each sampling point. The leaf area index (LAI) of wheat was measured by a LAI-2200C (Li-Cor, Inc.) plant canopy analyzer, and each sampling point was measured by two methods: across the ridge and parallel to the ridge. Each measurement was collected three times. Finally, the mean value was considered as the LAI value for each point.

### Data processing and analysis methods

#### Data processing

The interval method was used to select abnormal values. The interval is [μ-3 s, μ+3 s], μ is the mean value of the sample data, s is the standard deviation, and the outliers outside the interval are replaced by the normal maximum and minimum values.

#### Analysis methods

The eigenvalues of the classical statistical methods include minimum (min), maximum (max), mean, standard deviation (SD), skewness (Skew), kurtosis (Kurt) and coefficient of variation (CV), which can be used to describe the basic physical characteristics of soil nutrients. Among them, the CV is a parameter that can be used to compare the degree of data discretization under different measurement scales. According to the CV classification level, <10% indicates weakly variability, 10% to 100% indicates medium variability, and >100% indicates strong variability [31]. The single factor analysis of variance-least significant difference (LSD) method in SPSS software was used to analyze the significant differences in soil nutrients in different growth stages, and the level of confidence was 0.05.

The spatiotemporal variability of soil nutrients was mainly analyzed by a semivariogram. Moran’s I and fractal dimension (D) were combined to determine the spatial correlation and agglomeration degree at the same time. The formula for Moran’s I is as follows:

$$I=\frac{n}{\sum_{i}\sum_{j}\omega_{i,j}}\frac{\sum_{i}\sum_{j}\omega_{i,j}(x_{i}-\overset{-}{x})(x_{j}-\overset{-}{x})}{\sum_{i}{\left(x_{i}-\overset{-}{x}\right)}^{2}}$$

When calculating the value of Moran’s I, it usually gives a threshold that can determine whether to reject the null hypothesis, called Z. If the value of Z is in the range of -1.96–1.96, then P>0.05, indicating that null hypothesis of spatial randomness cannot be rejected; If the value of Z is out of this range (Z > 1.96 or Z < −1.96), then the spatial pattern cannot be the result of random process. In this case, the null hypothesis can be rejected, and the spatial distribution shown is a significant aggregation or dispersion pattern [32]. The value of Moran’s I is between -1 and 1. When the adjacent region has a similar attribute value, the value of Moran’s I is positive. When the adjacent area is different, Moran’s I is negative; when the attribute value exhibits pure randomness, Moran’s I tends to be 0. First, the AN, AP and AK data for each growth stage were input to Gs+ software, and the distribution point maps were obtained by a semivariogram. Then, the point maps were fitted with spherical, exponential, Gaussian and linear models. Finally, the best model and its corresponding parameter index were obtained. Among the results, the values of C_0_/(C_0_+C) represent the proportion of the variation caused by random factors to the total spatial variation and are often used as the basis of the classification of the spatial variation in variables. When C_0_/(C_0_+C) <25%, the system has a strong spatial correlation; when it is between 25% and 75%, the system has a moderate spatial correlation; when it is >75%, the system has a weak spatial correlation [33].

The spatial distribution map of soil AN, AP and AK in different growth stages was plotted using the ordinary kriging interpolation method. The classification standard for soil nutrients in the interpolation map was formulated according to China’s second national soil survey. AN was divided into 3 categories and 6 grades: high: I, >150 mg/kg, II, 120–150 mg/kg; medium: III, 90–120 mg/kg, IV, 60–90 mg/kg; low: V, 30–60 mg/kg, VI, <30 mg/kg. AP was divided into 3 categories and 7 grades: high: I, >100 mg/kg, II, 60–100 mg/kg; medium: III, 40–60 mg/kg, IV, 20–40 mg/kg, V, 10–20 mg/kg; low: VI, 5–10 mg/kg, VII, <5 mg/kg. AK was divided into 3 categories and 7 grades: high: I, >400 mg/kg, II, 350–400 mg/kg; medium: III, 300–350 mg/kg, IV, 250–300 mg/kg, V, 200–250 mg/kg; low: VI, 150–200 mg/kg, VII, <150 mg/kg. Based on the interpolated nutrient map, the professional viewpoints of landscape ecology were referenced, including 2 landscape level indices: patch number (NP) and patch density (PD) and 1 type level index: the percentage of landscape area (PLAND). A total of 3 indices were selected to quantitatively analyze the spatial dynamic characteristics of soil nutrient variation.

The values of SPAD and LAI at different growth stage of wheat and the soil AN, AP, and AK were compared at each point. The correlation coefficient was calculated using Pearson correlation analysis to analyze the response of winter wheat growth to soil nutrient changes.

## Results and analysis

### Descriptive statistical analysis of soil nutrients in different growth stages of winter wheat

The statistical characteristics of soil nutrients in different growth stages of winter wheat are shown in Table 1. The AN content ranged from 50.82 to 91.24 mg/kg, and the CV ranged from 51.50% to 56.47% from before the sowing stage to the filling stage. The AN content reached a turning point at the reviving stage. The AN content increased slightly before the reviving stage and then decreased significantly after the reviving stage. There was no significant difference in AN content between the before sowing stage and reviving stages, the jointing and filling stages, but the AN content in the before sowing and reviving stages was significantly higher than that during the jointing and filling stages. The AP content ranged from 35.70 to 64.37 mg/kg, and the CV ranged from 34.56% to 59.14% from the before sowing stage to the filling stage. The AP content was low before sowing and was significantly different from that in the other three growth stages. At the reviving stage, the AP content increased significantly and increased slightly at the jointing stage and then decreased slightly at the filling stage. The CV for AP content was moderate in each growth stage, but the CV before sowing appeared higher, followed by jointing stage, and the lowest values occurred at the reviving stage and filling stage. The AK content ranged from 285.19 to 377.13 mg/kg, and the CV ranged from 16.84% to 28.06% from the before sowing stage to the filling stage. The AK content in the reviving stage was significantly higher than that in the other stages. The AK content increased significantly from the before sowing stage to the reviving stage but decreased significantly at the jointing stage. The CV for AK was moderate in each growth stage, slightly higher before sowing, lower at the jointing stage, and moderate at the reviving stage and filling stage, but the differences were not significant.

**Table 1:** Statistical characteristics of soil nutrients during the different growth stages of winter wheat.

Index	Growth stage	Min (mg/kg)	Max (mg/kg)	Mean (mg/kg)	SD (mg/kg)	Skew	Kurt	K-S test	CV (%)	Significance	F
AN	B	15.29	213.27	88.73 a	45.06	0.66	0.01	0.42	51.50	0.00	22.01**
	R	13.83	259.25	91.24 a	55.71	0.71	0.25	0.37	56.47		
	J	3.82	116.95	54.45 b	28.09	0.37	-0.72	0.56	51.59		
	F	8.35	99.54	50.82 b	26.75	0.10	-1.25	0.21	55.89		
AP	B	4.90	95.28	35.70 b	20.39	0.96	0.93	0.47	59.14	0.00	24.91**
	R	16.94	102.48	59.49 a	20.56	0.29	-0.50	0.57	34.56		
	J	15.32	133.85	64.37 a	28.11	0.50	-0.38	0.66	44.53		
	F	16.70	109.70	57.37 a	19.62	0.62	0.59	0.36	34.95		
AK	B	125.79	474.4	285.19 b	80.02	0.07	-0.86	0.10	28.06	0.00	26.74**
	R	197.47	408.41	377.13 a	95.62	0.31	-0.24	0.95	25.22		
	J	170.11	406.14	299.52 b	50.43	-0.29	0.16	0.20	16.84		
	F	181.23	412.15	292.08 b	70.32	0.07	-1.21	0.57	24.08		

Note: AN: available N; AP: available P; AK: available K. B: before sowing; R: reviving stage; J: jointing stage; F: filling stage. The same is true below. The LSD method was used for multiple comparisons. The same letters in each column indicate that there were no significant differences in the average value. The difference was significant when the significance was <0.05,

**extremely significant.

In general, the soil nutrient contents showed the characteristics of low-high-low and moderate variability at the different growth stages. The AN and AK contents were highest in the reviving stage and AP was in the jointing stage. The AN content in the study area was relatively low, while the AP and AK contents were high according to the grading standard of China’s second national soil survey. The order of the degree of variation in each nutrient was AN>AP>AK. The results of the Kolmogorov-Smirnov test showed that under the 5% test standard, the AN, AP and AK of each growth stage obeyed a normal distribution after logarithmic transformation, which met the requirements for Moran’s I and kriging interpolation.

### The characteristics of soil nutrient variations in different growth stages of winter wheat

The semivariogram models and parameters for soil nutrients in different growth stages of winter wheat are shown in S1 Fig, Table 2. The best-fitting models for the three nutrients before sowing were all exponential models, indicating that each nutrient has a similar spatial variation trend and that the combination of the characteristics of soil nutrient variations was the simplest throughout the growth period. The best-fitting model for AN was the exponential model, while the best-fitting model for AP and AK was the spherical model at the reviving stage. The variation trends of AP and AK were similar but different from that of AN in this stage. The combination of the characteristics of soil nutrient variations was the most complicated in the jointing stage, which is contrary to that observed before sowing. The best-fitting models of AN, AP and AK were spherical, exponential and Gaussian models, respectively. In the filling stage, the Gaussian model was best-fitting model for AN and AP, and the best-fitting model for AK was the exponential model, which showed that the spatial variation trends of AN and AP were similar. However, different from AK, the characteristics of the soil nutrient variations during the filling stage and the reviving stage were similar in complexity to those before sowing and during the jointing stage.

**Table 2:** Semivariogram theoretical models and parameters of soil nutrients during the different growth stages of winter wheat.

Growth stage	Index	Model	C_0_	C_0_+C	C_0_/(C_0_+C) (%)	A (m)	R^2^	RMSSE	D	*I*	Z
B	AN	E	0.0060	0.0583	10.29	245.7	0.941	1.68×10^−5^	1.857	0.305	2.65
	AP	E	0.0073	0.0844	8.65	164.7	0.993	2.13×10^−6^	1.897	0.226	3.42
	AK	E	0.0024	0.0192	12.5	235.2	0.976	7.79×10^−7^	1.817	0.320	2.22
R	AN	E	0.0408	0.1055	38.67	179.8	0.969	2.10×10^−5^	1.887	0.282	2.44
	AP	S	0.0090	0.0262	34.35	142.5	0.920	3.08×10^−6^	1.925	0.209	1.99
	AK	S	0.0036	0.0138	26.09	235.8	0.997	8.70×10^−8^	1.914	0.153	4.26
J	AN	S	0.0233	0.0860	27.09	146.8	0.913	4.92×10^−5^	1.855	0.280	2.85
	AP	E	0.0035	0.0419	8.35	122.7	0.907	5.49×10^−6^	1.900	0.212	2.63
	AK	G	0.0007	0.0065	10.77	108.1	0.982	3.36×10^−8^	1.853	0.260	2.02
F	AN	G	0.0333	0.0916	36.35	127.5	0.904	6.93×10^−5^	1.890	0.270	1.98
	AP	G	0.0048	0.0302	15.89	88.2	0.938	2.20×10^−6^	1.941	0.177	2.03
	AK	E	0.0017	0.0123	13.82	117.9	0.929	2.72×10^−7^	1.934	0.125	2.26

Note: E: exponential model; S: spherical model; G: Gaussian model. D: the fractal dimensions. *I*: Moran’s I.

In general, the semivariogram of the three nutrients were represented by the same model at the before sowing stage, two models at the reviving stage, three models at the jointing stage, and then two models at the filling stage. The characteristics of the soil nutrient variations were greatest at the jointing stage, and the difference first increased and then decreased. The R^2^ of the best-fitting model for each nutrient ranged from 0.904 to 0.997, and the root mean square standardized error (RMSSE) ranged from 3.36×10^−8^ to 6.93×10^−5^, which indicated that the selected model of the semivariogram was optimal and could adequately reflect the characteristics of the spatial structure and variation trends of soil nutrients throughout the growth period.

The C_0_ values of AN, AP and AK in the four growth stages were all close to 0, indicating that the variation caused by the test and sampling errors was very small. The C_0_/ (C_0_+C) of AN was 10.29% before sowing with strong spatial correlation, and it ranged from 27.09% to 38.67% from the reviving stage to the filling stage, with moderate spatial correlation. The C_0_/ (C_0_+C) values of AP at the before sowing, jointing and filling stages were 8.65%, 8.35% and 15.89%, respectively, with strong spatial correlation, and this value was 34.35% at the reviving stage with moderate spatial correlation. The C_0_/ (C_0_+C) values of AK at the before sowing, jointing and filling stages were 12.5%, 10.77 and 13.82%, respectively, with strong spatially correlated, and this value was 26.09% at the reviving stage with moderate spatial correlation. Overall, the three nutrients showed moderate to strong spatial correlation with the progression of the growth stages and experienced a process of weakening-strengthening-weakening. These three nutrients were strongly spatially correlated before sowing, and the spatial correlation was moderate during the reviving stage. For the jointing and filling stages, AN exhibited moderate spatial correlation, and AP and AK were strongly spatially correlated. These three nutrients exhibited the weakest spatial correlation at the reviving stage, the spatial correlation of AN was strongest in the before sowing stage, while the spatial correlations of AP and AK were the strongest at the jointing stage.

The fractal dimensions of the three nutrients were from 1.817–1.897, 1.887–1.925, 1.853–1.900 and 1.890–1.941 from the before sowing stage to the filling stage, indicating that the soil nutrients in each growth stage had good fractal characteristics and structure. The fractal dimension values showed a process of first increasing then decreasing and then increasing from the before sowing stage. This result indicated that the structure of the soil nutrients became more complex, and the spatial correlation was weakened from the before sowing stage to the reviving stage and from the jointing stage to the filling stage, while the structure of nutrients tended to be consistent, and the spatial correlation increased from the reviving stage to the jointing stage.

The Z values of AN, AP and AK in the four growth stages were all >1.96, indicated that the null hypothesis of spatial randomness could be rejected and the spatial distribution was a significant aggregation pattern. The spatial autocorrelation are shown in S2 Fig. Moran’s I of the three nutrients ranged from 0.226–0.320, 0.153–0.282, 0.212–0.280 and 0.125–0.270 from the before sowing stage to the filling stage. The value of Moran’s I showed a process of first decreasing then increasing and then decreasing from the before sowing stage. The results showed that the soil nutrient structure gradually dispersed, and the spatial correlation decreased from the before sowing stage to the reviving stage and from the jointing stage to the filling stage. The structure of the nutrients tended to gather, and the spatial correlation increased from the reviving stage to the filling stage.

In general, as the growth progressed, (1) each nutrient showed moderate to strong spatial correlation. (2) The three nutrients exhibited strong spatial correlation during the before sowing stage and moderate spatial correlation during the reviving stage. For the jointing and filling stages, AN exhibited a moderate spatial correlation, and AP and AK exhibited strong spatial correlation. (3) The weakest spatial correlation of the three nutrients was shown at the reviving stage; the strongest spatial correlation of AN was shown before sowing, while the AP and AK exhibited the strongest spatial correlation at the jointing stage. (4) The spatial correlation of soil nutrients decreased from before sowing to the reviving stage, jointing stage and filling stage, and increased from the reviving stage to the jointing stage.

### The spatial distribution characteristics of soil nutrients in different growth stages of winter wheat

**Fig 2:**
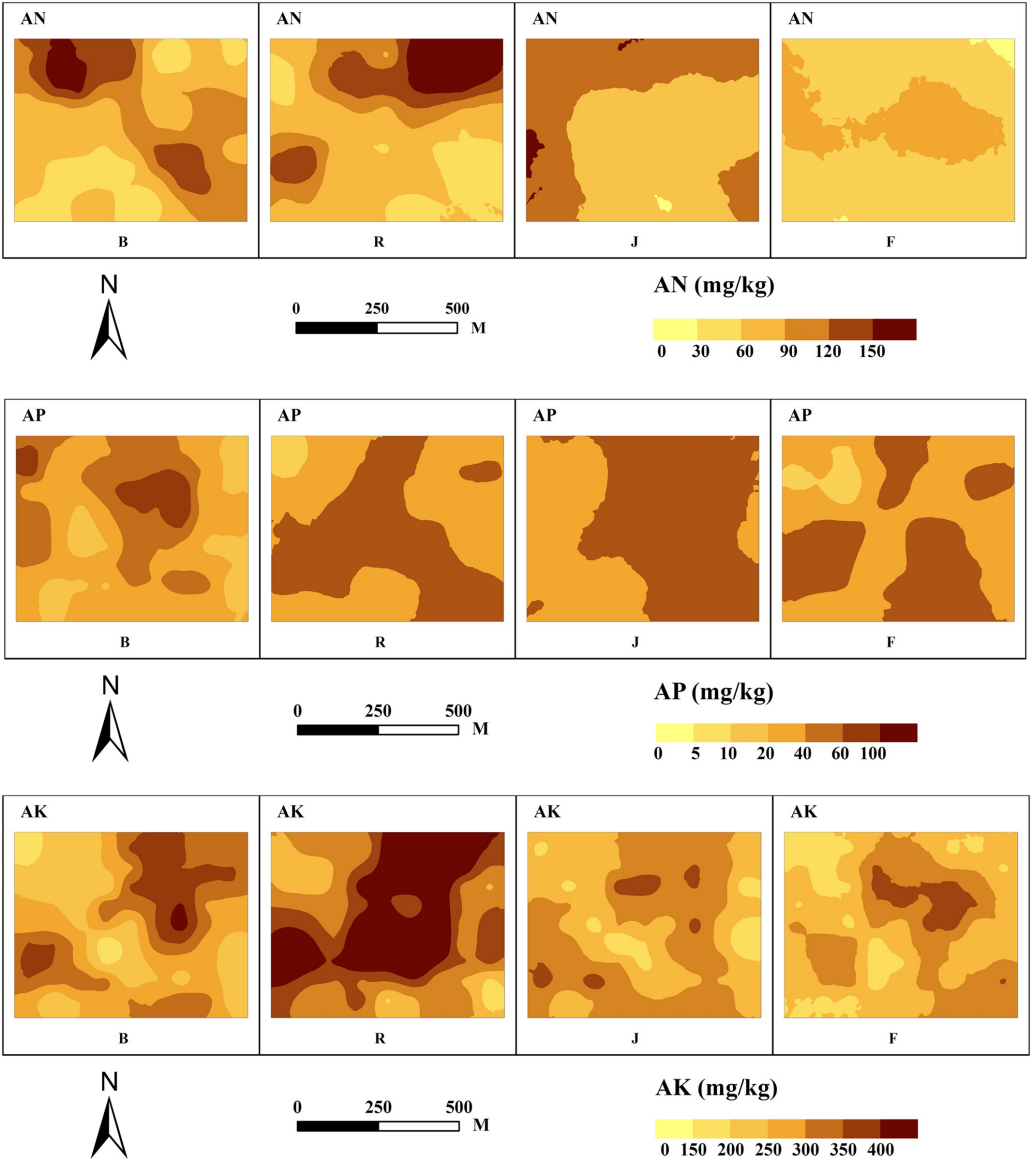
Spatial distribution of soil nutrients during the different growth stages of winter wheat. Note: AN: available N; AP: available P; AK: available K. B: before sowing; R: reviving stage; J: jointing stage; F: filling stage.

The spatial and temporal distribution maps of soil nutrients in the different growth stages of winter wheat are shown in Fig 2. The distribution of AN before sowing showed a striped pattern, and the content from northwest to southeast was obviously higher than that in other regions. For the reviving stage, the concentration of AN declined slightly, and the content in the study area was opposite to that before sowing. The areas with high AN contents were concentrated in the northeast to southwest areas. For the jointing stage, AN showed a clustered distribution, and the AN content was significantly decreased, mainly concentrated from 60~90 mg/kg. For the filling stage, the continuity of the distribution continued to increase, and the AN content continued to decline, reaching a value lower than 60 mg/kg. The distribution of AP before sowing was dispersed, and there were no clear centers of high and low contents. For the reviving stage, the distribution of AP showed a striped pattern, and the AP content increased significantly at this stage. For the jointing stage, the boundaries of the high and low regions were obvious, the overall trend was high in the east and low in the west, and the AP content was high throughout the study area, mainly concentrated at > 60 mg/kg. For the filling stage, AP showed a clustered distribution, and the area with high nutrient contents decreased. The distribution of AK before sowing showed a clustered distribution, and the high content area was concentrated in the northeast. For the reviving stage, the distribution of AK showed a striped pattern, and the area with high contents increased. For the jointing stage, the continuity of the AK distribution decreased significantly, and the area with high contents decreased rapidly. For the filling stage, the distribution characteristics of AK were similar to those at the jointing stage; the AK content decreased further, and the area with low contents increased.

The landscape pattern indices for the interpolation of soil nutrients in different growth stages of winter wheat are shown in Tables 3, 4 and 5. The distribution of AN was consistent at the landscape level during the different stages. Patch number and PD showed the characteristics of increased-decreased-decreased. From before sowing to the filling stage, the proportions of the areas that showed high grades (I and II) were 20.39%, 28.32%, 0%, and 0%, the proportions of the areas that showed middle graded (III and IV) were 55.79%, 50.56%, 49.33%, and 27.6%, and the proportions of the areas that showed low graded (V and VI) were 23.82%, 21.11%, 50.66%, and 72.4%. This result showed that with the progression of the growth stages, the proportions of the area with high and low levels of AN exhibited an opposite trend, and the proportion of the area with high AN content gradually decreased after the reviving stage, while the proportion of the area with low grades increased. The distribution of AP was consistent at the landscape level during the different stages. Patch number and PD showed decreased-decreased-increased characteristics. From before sowing to the filling stage, the proportions of the area with high grades (I and II) were 10.18%, 55.23%, 67.81%, and 48.77%, the proportions of the area with middle grades (III, IV, V) were 88.28%, 44.77%, 32.17%, and 51.23%, and the proportions of the areas with low grades (VI and VII) were 1.53%, 0%, 0%, and 0%. This result showed that the dominant areas were converted from high contents to moderate contents. The distribution of AK was consistent at the landscape level during the different stages and showed a characteristic of continuous increase. From before sowing to the filling stage, the proportions of the area with high grades (I and II) were 18.13%, 55.59%, 6.38%, and 6.34%, the proportions of the area with middle grades (III, IV, V) were 76.4%, 44.41%, 93.62%, and 92.56%, and the proportions of the area with low grades (VI and VII) were 5.46%, 0%, 0%, and 1.1%. This result showed that with the progression of the growth stages, the AK at the type level exhibited changes that were similar to the changes in AN in general, and the proportion of the area with high grades decreased after the reviving stage. Generally, throughout the growth stages of winter wheat, the contents of soil nutrients first increased and then gradually decreased, while the number of nutrient grades, especially the number of high content grades, gradually decreased.

**Table 3:** Variation in AN landscape indices at the landscape and class level during the different growth stages of winter wheat.

Growth stage	Index	Class I	Class II	Class III	Class IV	Class V	Class VI
B	NP	1	2	1	5	3	1
	PD (/hm^2^)	0.0286	0.0571	0.0286	0.1429	0.0857	0.0286
	PLAND	4.52%	15.87%	18.56%	37.23%	22.87%	0.95%
R	NP	1	2	2	6	3	
	PD (/hm^2^)	0.0286	0.0571	0.0571	0.1714	0.0857	
	PLAND	14.38%	13.94%	16.55%	34.01%	21.11%	
J	NP			5	2	1	1
	PD (/hm2)			0.1429	0.0571	0.0286	0.0286
	PLAND			1.76%	47.57%	50.23%	0.43%
F	NP				2	4	2
	PD (/hm2)				0.0571	0.1143	0.0571
	PLAND				27.60%	71.27%	1.13%

Note: NP: patch number; PD: patch density; PLAND: percentage of landscape area. The same is true below.

**Table 4:** Variation in AP landscape indices at the landscape and class level during the different growth stages of winter wheat.

Growth stage	Index	Class I	Class II	Class III	Class IV	Class V	Class VI	Class VII
B	NP		2	3	2	5	1	1
	PD (/hm^2^)		0.0571	0.0857	0.0571	0.1429	0.0286	0.0286
	PLAND		10.18%	31.96%	39.11%	17.21%	0.81%	0.72%
R	NP	1	3	3	1			
	PD (/hm^2^)	0.0286	0.0857	0.0857	0.0286			
	PLAND	0.92%	54.31%	41.38%	3.39%			
J	NP		2	3				
	PD (/hm^2^)		0.0571	0.0857				
	PLAND		67.81%	32.17%				
F	NP		4	1	1			
	PD (/hm^2^)		0.1143	0.0286	0.0286			
	PLAND		48.77%	45.35%	5.88%			

**Table 5:** Variation in AK landscape indices at the landscape and class level during the different growth stages of winter wheat.

Growth stage	Index	Class I	Class II	Class III	Class IV	Class V	Class VI	Class VII
B	NP	1	2	3	1	4	2	1
	PD (/hm^2^)	0.0286	0.0571	0.0857	0.0286	0.1143	0.0571	0.0286
	PLAND	1.83%	16.30%	21.99%	26.13%	28.28%	3.95%	1.51%
R	NP	2	3	3	4	2		
	PD (/hm^2^)	0.0571	0.0857	0.0857	0.1143	0.0571		
	PLAND	33.87%	21.72%	30.19%	12.86%	1.36%		
J	NP	1	5	1	3	6		
	PD (/hm^2^)	0.0286	0.1429	0.0286	0.0857	0.1714		
	PLAND	1.24%	5.14%	55.85%	31.46%	6.31%		
F	NP		2	2	7	9	1	
	PD (/hm^2^)		0.0571	0.0571	0.2	0.2571	0.0286	
	PLAND		6.34%	31.16%	47.27%	14.13%	1.10%	

### Correlation between soil nutrients and wheat growth during the same growth stage and subsequent stage

The correlation coefficients between soil nutrients and the index of wheat growth are shown in Table 6. There were good correlations between the LAI of wheat and AN, AP, AK and SPAD. Among them, SPAD, LAI and AN reached significant positive correlations at each growth stage. There was not a significant correlation between SPAD and AP at any growth stage. There was a significant correlation between LAI and AP at the filling stage, but there was no correlation during the period from the reviving stage to the jointing stage. There was a significant correlation between SPAD and AK at the filling stage, but there was no correlation during the period from the reviving stage to the jointing stage. There was no correlation between LAI and AK at the filling stage, but it reached a significant correlation during the period from the reviving stage to the jointing stage.

**Table 6:** Correlation coefficients between soil nutrients and wheat growth stage.

Growth stage	Index	AN	AP	AK
R	SPAD	0.2823*	0.146	0.1683
	LAI	0.2097*	0.1147	0.1426
J	SPAD	0.5007**	0.1598	0.2063*
	LAI	0.5412**	0.1737	0.2587*
F	SPAD	0.5168**	0.1956	0.2136*
	LAI	0.5635**	0.2025*	0.2645*

Note:

**P<0.01;

*P<0.05. The same is true below.

With the increase or decrease in AN content in the soil, the SPAD and LAI values also increased or decreased correspondingly during the period from the reviving stage to the filling stage. With the increase in AP content in the soil, the LAI values also increased correspondingly at the filling stage, while the change in SPAD at the filling stage and the changes in SPAD and LAI at the other three stages were not obvious. These results showed that AP had different effects on wheat growth at different growth stages. The SPAD and LAI values were significantly correlated with the AK content in the soil during the jointing stage to the filling stage, and the correlation coefficients between these two growth stages were not significantly different. This result indicated that AK played an important role during the late growth stage of wheat and had a steady effect on wheat growth compared with AN and AP.

The correlation coefficients between the soil nutrient contents at the current stage and the index of wheat growth at the following growth stage are shown in Table 7. There was also a good correlation between the soil nutrient content at the current stage and the index of wheat growth at the following growth stage. There were extremely significant correlations between AN (before sowing) and SPAD (reviving stage), AN (jointing stage) and SPAD (filling stage), LAI (filling stage). There were significant correlations between AN (before sowing) and LAI (reviving stage), AK (before sowing) and SPAD (reviving stage), AK (jointing stage) and SPAD, and AK (jointing stage) and LAI (filling stage).

**Table 7:** Correlation coefficients between soil nutrients and wheat growth stage of the following growth stage.

Growth stage	Index	SPAD-2	LAI-2
R	AN	0.3204**	0.2722*
	AP	0.183	0.0964
	AK	0.2049*	0.1874
J	AN	0.1714	0.1241
	AP	0.045	0.084
	AK	0.1158	0.045
F	AN	0.461**	0.4922**
	AP	0.1804	0.1541
	AK	0.2561*	0.2241*

Note: XX-2 is the index of winter wheat growth in the following stage;

**P<0.01;

*P<0.05.

The comparison of Tables 6 and 7 indicates that the correlation coefficients between AN, AP and AK (before sowing) and the SPAD and LAI (reviving stage) values were significantly higher than the correlation coefficients between AN, AP and AK (reviving stage) and the SPAD and LAI values (reviving stage). This result indicated that the nutrients before sowing had a more obvious effect on the growth of wheat at the reviving stage than the nutrients at the reviving stage. The correlation coefficients between AN, AP and AK (reviving stage) and the SPAD and LAI values (jointing stage) were significantly lower than the correlation coefficients between AN, AP and AK (jointing stage) and the SPAD and LAI values (jointing stage). This result indicated that the growth of wheat at the jointing stage was mainly affected by the nutrients of the current stage, which was related to the jointing stage being a peak period of nutrient uptake by wheat. This finding was mainly related to the reason that the jointing stage is a peak period of soil nutrient absorption by wheat. The correlation coefficients between AN, AP and AK (jointing stage) and the SPAD and LAI values (filling stage) were significantly lower than the correlation coefficients between AN, AP and AK (filling stage) and the SPAD and LAI values (filling stage), but it still reached significant correlation. This result indicated that the growth of wheat at the filling stage was affected by the soil nutrients at both the jointing stage and filling stage.

In general, the growth status of wheat was closely related to the nutrients in the soil. AN had the highest correlation with wheat growth status, followed by AK and AP. At the reviving stage, the effect of nutrients before sowing on the growth of wheat was slightly higher than the effect of nutrients in the reviving stage. At the jointing stage, the effect of nutrients in the jointing stage on the growth of wheat was much higher than the effect of nutrients in the reviving stage. At the filling stage, the status of wheat growth was affected by the nutrients in the jointing stage and filling stage.

## Discussion

(1) Many experimental design methods have the advantages of easy access to data, low cost and small interference, but these conditions are different from the real field environment and management mode, and the conclusions of these studies have great limitations in the application of agricultural extension. Based on this, this research studied the soil nutrients and winter wheat under the traditional tillage model, and the conclusions were more practical and could be more suitable for winter wheat planting areas in northern China.

(2) Huantai County is located in the Huanghuai winter wheat area, which is the main wheat production area of China where the planting conditions are superior. It was representative to study the variation in soil nutrients and their correlation with crop growth during the winter wheat growth period. The minimum spatial correlation distance of soil nutrients in each growth stage was 88.2 m, which exceeded the sampling interval (60 m) and indicated that the sampling interval was reasonable. In this study, the dynamic changes in soil nutrient content during the winter wheat growth period were generally consistent with the results of research conducted in Jiangyan City, Jiangsu Province, China [34].

(3) Soil nutrients showed moderate variations during the growth stage of winter wheat, generally showing that the concentrations of AN were slightly higher than those of AP but much larger than those of AK. The reason for this difference was mainly related to fertilization. The high variability of AN was due to the large demand for nitrogen by wheat, and farmers pay great attention to the use of urea. The high variability of AP was related to the chemical behavior of P elements in the soil [35]. The mobility of AP was weak, and its utilization rate was low, resulting in a large amount of fertilizer residue in the soil and uneven distribution, which was also confirmed by the maximum variation in AP before sowing in this research. The soil parent material in the study area was rich in potassium and was in excess of that required for the growth of wheat. Therefore, the influence of field management on AK was small, so the variability was low.

(4) Single C_0_/ (C_0_+C) values were not stable or effective in describing the structure of the variation when the C_0_/ (C_0_+C) values were large in a small area [36]. Therefore, a method of mutual verification of C_0_/ (C_0_+C), fractal dimension and Moran’s I was adopted in this research. The results showed that the structure of soil nutrients weakened from before sowing to the reviving stage and from the jointing stage to the filling stage and tended to strengthen from the reviving stage to the jointing stage. This pattern may be caused by field management and nutrient absorption during wheat growth. In particular, urea supplementation at the reviving stage and irrigation at the filling stage were the main reasons for the obvious decreases in soil nutrient correlation. The nutrient uptake of winter wheat peaked at the jointing stage, and the soil nutrient distribution tended to be balanced and then structurally enhanced. Significant changes in soil nutrient correlation were also found during the growth period of tobacco [37]. Wheat is one of the most important food crops in the world; thus, systematically studying the effects of wheat growth on soil nutrients was of great significance. However, the results in this field still need to be enriched.

(5) The correlation coefficients between AN and SPAD and LAI values were the highest at all growth stages of winter wheat, followed by those for AK and AP. This result may be related to the differences in the demands for different nutrients during the growth stage of winter wheat. The ratio of nitrogen, phosphorus and potassium absorption in wheat was 3.1:1:3.14 in the brown soil area of western Henan Province, China [38], which has planting conditions similar to those in this research area. This finding was basically consistent with the conclusions of this study. The correlation between the soil nutrients and the growth index of the following growth stage showed that the growth of the winter wheat in the reviving stage was mainly influenced by the soil nutrients in the previous stage (before sowing), indicating that there was a temporal delay from fertilization to the impacts of fertilizer on crop growth. The effects of soil nutrients at the jointing stage on the growth of wheat at the jointing stage were much higher than the effects of soil nutrients in the jointing stage. This result was related to the high demands for fertilizer at the jointing stage of wheat [39]. Therefore, ensuring the supply of fertilizer at the jointing stage is of great significance. At the filling stage, the effect of nutrients in the previous stage (jointing stage) on the growth of winter wheat was obvious. Because the traditional period of urea supplementation is generally during the reviving stage in northern China, in view of the importance of the jointing stage to the growth of winter wheat, the time of fertilizer supplementation should be properly moved to the jointing stage to ensure the nutrient supply in the later stage of wheat growth. The spatial and temporal distributions of more biological indicators of winter wheat need to be further studied.

## Conclusion

(1) The contents of three nutrients all showed the characteristics of decreased-increased-decreased and moderate variability. The AN content reached a turning point at the reviving stage, while the AP and AK contents reached turning points at the jointing stage. AN had the largest CV, followed by AP and AK.

(2) The three nutrients all showed medium to strong spatial correlation. The three nutrients showed the weakest spatial correlation at the reviving stage; the strongest spatial correlation of AN occurred before sowing, while the strongest spatial correlations of AP and AK were at the jointing stage. The correlation of soil nutrients decreased from before sowing to the reviving stage, from the jointing stage to the filling stage, and increased from the reviving stage to the jointing stage.

(3) The high AN contents gradually decreased, and the spatial distribution continuously increased; the high AP contents decreased slightly, the change in the spatial distribution continuity was not obvious; the high AK contents significantly decreased, and the spatial distribution continuity continuously decreased.

(4) AN exhibited the highest correlation with wheat growth status, followed by AK and AP. At the reviving stage, the effect of nutrients before sowing on the growth of wheat was slightly higher than that at the reviving stage. At the jointing stage, the effect of the nutrients in the jointing stage on the growth of wheat was much higher than that in the reviving stage. At the filling stage, the status of wheat growth was affected by the nutrients in both the jointing stage and filling stage.

The temporal and spatial characteristics of the soil nutrient variations and the response of wheat growth throughout the winter wheat growth period under the traditional tillage model were clearly described, which could provide a scientific basis for the effective guidance for wheat production and the popularization of precision fertilization technology.

## Supporting information

S1 Fig Semivariogram theoretical models of soil nutrients in different growth stages of winter wheat. (DOCX)

S2 Fig Spatial autocorrelation of soil nutrients in different growth stages of winter wheat. (DOCX)
