# Supplementary material for: Spatiotemporal variability of soil nutrients and the responses of growth during growth stages of winter wheat in northern China
Source: PLoS One. 2018 Dec 4;13(12):e0203509. doi: 10.1371/journal.pone.0203509 (PMC6279044; doi:10.1371/journal.pone.0203509)
Supplement: S1 Fig — (DOCX) [file pone.0203509.s001.docx]

**Supplementary materials**

**Spatiotemporal variability of soil nutrients and the responses of growth during growth stages of winter wheat in northern China**

**Baowei Su^1^, Gengxing Zhao^1^*, Chao Dong^2^**

^1^ College of Resources and Environment, Shandong Agricultural University, Tai’an, Shandong 271018, China,

^2^ College of Information Science and Engineering, Shandong Agricultural University, Tai’an, Shandong 271018, China.

*Corresponding author: E-mail: [zhaogx@sdau.edu.cn](mailto:zhaogx@sdau.edu.cn)

**S1 Fig. Semivariogram theoretical models of soil nutrients in different growth stages of winter wheat.**

**The semivariogram theoretical models of AN during before sowing stage**

**The semivariogram theoretical models of AP during before sowing stage**

**The semivariogram theoretical models of AK during before sowing stage**

**The semivariogram theoretical models of AN during reviving stage**

**The semivariogram theoretical models of AP during reviving stage**

**The semivariogram theoretical models of AK during reviving stage**

**The semivariogram theoretical models of AN during jointing stage**

**The semivariogram theoretical models of AP during jointing stage**

**The semivariogram theoretical models of AK during jointing stage**

**The semivariogram theoretical models of AN during filling stage**

**The semivariogram theoretical models of AP during filling stage**

**The semivariogram theoretical models of AK during filling stage**
