# Supplementary material for: Spatiotemporal variability of soil nutrients and the responses of growth during growth stages of winter wheat in northern China
Source: PLoS One. 2018 Dec 4;13(12):e0203509. doi: 10.1371/journal.pone.0203509 (PMC6279044; doi:10.1371/journal.pone.0203509)
Supplement: S2 Fig — (DOCX) [file pone.0203509.s002.docx]

**Supplementary materials**

**Spatiotemporal variability of soil nutrients and the responses of growth during growth stages of winter wheat in northern China**

**Baowei Su^1^, Gengxing Zhao^1^*, Chao Dong^2^**

^1^ College of Resources and Environment, Shandong Agricultural University, Tai’an, Shandong 271018, China,

^2^ College of Information Science and Engineering, Shandong Agricultural University, Tai’an, Shandong 271018, China.

*Corresponding author: E-mail: [zhaogx@sdau.edu.cn](mailto:zhaogx@sdau.edu.cn)

**S2 Figure Spatial autocorrelation of soil nutrients in different growth stages of winter wheat.**

**Spatial autocorrelation of AN during before sowing stages**

**Spatial autocorrelation of AP during before sowing stages**

**Spatial autocorrelation of AK during before sowing stages**

**Spatial autocorrelation of AN during reviving stages**

**Spatial autocorrelation of AP during reviving stages**

**Spatial autocorrelation of AK during reviving stages**

**Spatial autocorrelation of AN during jointing stages**

**Spatial autocorrelation of AP during jointing stages**

**Spatial autocorrelation of AK during jointing stages**

**Spatial autocorrelation of AN during filling stages**

**Spatial autocorrelation of AP during filling stages**

**Spatial autocorrelation of AK during filling stages**
